# Biological Activities of Grape Seed By-Products and Their Potential Use as Natural Sources of Food Additives in the Production of Balady Bread

**DOI:** 10.3390/foods11131948

**Published:** 2022-06-30

**Authors:** Haiam O. Elkatry, Abdelrahman R. Ahmed, Hossam S. El-Beltagi, Heba I. Mohamed, Nareman S. Eshak

**Affiliations:** 1 Food and Nutrition Science Department, Agricultural Science and Food, King Faisal University, P.O. Box 400, Al-Ahsa 31982, Saudi Arabia; helkatary@kfu.edu.sa; 2 Home Economics Department, Faculty of Specific Education, Ain Shams University, Abassia, Cairo 11772, Egypt; 3 Agricultural Biotechnology Department, College of Agricultural and Food Science, King Faisal University, P.O. Box 400, Al-Ahsa 31982, Saudi Arabia; 4 Biochemistry Department, Faculty of Agriculture, Cairo University, Giza 12613, Egypt; 5 Biological and Geological Sciences Department, Faculty of Education, Ain Shams University, Cairo 11341, Egypt; hebaibrahim79@gmail.com; 6 Home Economics Department, Faculty of Specific Education, Assiut University, Assiut 71515, Egypt; nariman_saeed@aun.edu.eg

**Keywords:** balady bread, grape seed, production, polyphenols, flavonoids, antioxidant activity

## Abstract

The biological function of bioactive compounds found in plant by-products has triggered expanded interest in recent years. This study aims to produce balady bread enriched with dietary fiber, mineral, and phenolic compounds by the addition of grape seeds powder (GSP) at different levels (5%, 10%, and 15% as a partial substitute for wheat flour). The results show that balady bread (Bb) and grape seed powder have ash contents of about 1.97% and 3.04%, lipid contents of 3.22% and 17.15%, protein contents of 11.16% and 12.10%, fiber contents of 1.06% and 44.90%, and carbohydrates contents of 56.52% and 29%, respectively. Moreover, grape seed powder contains a higher level of iron and zinc about 30.02 and 9.43 mg/kg than the Bb control sample which contains about 8.19 and 7.25 mg/kg respectively. The findings revealed that balady bread fortified with grape seed powder contains a high amount of total polyphenols content (TPC), total flavonoid content (TF), and antioxidant capacity. The farinograph test results showed that increasing the GSP concentration in the flour above 10% reduced dough development, stability, and farinograph quality number. The addition of GSP to wheat flour accelerated the dough’s water absorption and mixing tolerance. Grape seed incorporation levels up to 10% (*w*/*w*) had no negative effect on dough rheological performance. The sensory evaluation of bread showed that samples that were enriched with grape seeds powder at up to 10% had good quality. Based on these findings, it is recommended to replace up to 10% GSP in the manufacturing of fortified balady bread with satisfactory physical and sensory characteristics and high TPC and antioxidant activity.

## 1. Introduction

The agro-food industry now produces a large amount of by-products that may contain added value compounds with high functionality and/or bioactivity. Furthermore, consumer demand for healthier foodstuffs has increased in recent years, and the food industry has worked hard to meet this challenge. By-products are generally secondary products derived from primary agro-food production processes, and they represent an interesting and less expensive source of potentially functional ingredients such as peptides, carotenoids, and phenolic compounds, promoting the circular economy concept [1,2,3,4,5,6]. Food waste resulting from food manufacturing processes in the world causes a lot of environmental problems that lead to many diseases for humans and animals as well as harm the planet, such as increasing global warming and pollution [7] as well as the high economic cost of disposing of such waste and its impact on the environment. Despite rising food demand, Egypt has a high rate of food loss and waste, particularly for perishable products. Fruit and vegetable waste is estimated to account for 45–55% of production in Egypt each year. Therefore, attention must be paid to the fruits and vegetable by-products and to make maximum use of them in order to contribute to solving these problems [8]. Plant by-products comprise oligosaccharides, phenolic compounds, proteins, as well as other compounds, making them a rich source of bioactive substances that can feasibly be used as sources of food ingredients in the food industry at no supplemental manufacturing cost and at lowered industrial costs [9,10]. Some artificial ingredients could alter human enzymes and lipids and may cause carcinogenic effects [11]. As a result, recent studies have been conducted to replace artificial ingredients with natural ingredients derived from agricultural waste [12]. The growing interest in natural antioxidant, antitumor, and antimicrobial substances has provided new insights into plant by-products as a source of these bioactive substances [13]. 

Consumers today are interested not only in food items that are safe or nutritious, but also in natural, edible, or healthy foods. Increased consumer interest in functional foods has resulted in an increase in demand for natural foods [14]. For certain food ingredients that are still in use, reusing fruit waste or by-products can provide a sustainable source or even produce new artificial additives with active substances and properties that will bring benefits to the food system [15]. Among the fruits, grapes are gaining momentum as a functional food element in the world [16]. Grapes are one of the most commonly grown fruits in the world, with a yearly output of approximately one million metric tons [17]. Each year, the winery processing industry generates large quantities of waste (pomace, seeds, and stems, among others), accounting for 13.5–14.5% of the overall volume of output. This bio-residue is generally used as fertilizers or dismissed [18]. In addition, they contain higher concentrations of polyphenols, tocopherols, and other macro- and micronutrients that tend to make winery waste an important and worthwhile raw material to gain these substances and provide them with added-value substances for bioactive uses [17,18]. The phenolic compounds of grapes are valuable phytochemicals because of their antioxidant and antimicrobial characteristics. These types of chemicals are made up of a diverse variety of different chemical compositions with strong biological activities that are found in every part of the grape, especially in the skin and seeds [18,19]. According to some estimates, about 20% of the total weight of grapes is waste in the form of seeds, stems, and skins, which translates into more than 8 million tons of waste in the world [20]. The remaining waste is about 25% of seeds, 25% of stems, and 50% of the peel. Removing this waste is expensive, and if not treated effectively, it is a serious environmental problem [21].

Grape seeds contain a high concentration of antioxidant compounds [22]. It contains a lot of phenolic compounds such as gallic acid, monomeric phenolic compounds such as epicatechin and catechin, and polymeric and oligomeric procyanidins [23]. In grape seeds, polyphenol compounds are 60–70%, compared to only 10% in fruits and 28–35% in peels [24]. Polyphenols have been linked to improved antioxidant properties, cardiovascular effects, anti-inflammatory effects, anti-inflammatory activity, antiviral, and anticancer activity [25,26]. Grape seed contains many flavonoids and anthocyanins as functional components that have antioxidant activity in the body and are important for preventing tissue oxidation damage by reducing fat oxidation or preventing and/or limiting the formation of free radicals [27].

Grape seed is added to wheat flour to enhance its chemical and rheological characteristics and thus improve the quality of baked products and increase their acceptance by the consumer [28]. Grape seeds are secondary products that can be considered extremely good ingredients for food products depending on the chemical constituents such as oil, protein, fiber, and sugar; they are also a great resource of minerals such as calcium, phosphorous, potassium, and iron [29]. Several studies have concentrated on the use of grape products in baked products such as bread, muffins, cakes [30], biscuits [31], and cookies [32], and to determine the specific and sensory properties of these products.

The concept of functional foods began to spread in the mid-eighties in Japan [33], and they are those foods that are supported by healthy active compounds and that bring health benefits to the human body other than the recognized nutritional value and have the same traditional sensory properties and maybe better. In recent years, the demand for these foods has increased, which has led to an increase in their production in factories [34,35,36]. Natural and functional nutrition has become the main priority among consumers because of its positive effects on health [37,38]. 

Bread is a staple food that is closely related to people’s daily life. The fortified bread is a good example of a functional food fortified with many bioactive compounds such as vitamins, minerals, fiber, and antioxidants because of its widespread use as a major source of energy [39]. Bread making is one of the oldest traditional industries throughout the ages, dating back to 10,000–12,000 BC, which was limited to mixing grain flour with water. It is believed that the Egyptians were among the first to develop this industry globally [40]. It is the primary source of carbohydrates and the primary dietary component for both rich and poor Egyptian consumers [41]. Balady bread is one of the most important components of the Egyptian diet, and as the population increased, it became very important to produce safe, whole grains for the bread. The Egyptian government supports the production of balady bread, which is made from wheat flour and contains 10 to 12%protein [42]. In terms of production and consumption, wheat is the world’s most important cereal crop [43]. Significant progress has been made in baking technology and changing food habits in recent years. Nowadays, due to the problem of growing wheat obtaining it. It must replace in the production of bread some amounts of wheat flour with important by-products such as grape seeds.

This study focused on the effect of grape seed powder integration as a natural antioxidant, dietary fiber, and sources of mineral on the physical, chemical, dough rheological and sensory properties of balady bread.

## 2. Materials and Methods

### 2.1. Materials

Wheat flour (72% extraction rate), sugar, fresh yeast, salt, corn oil, and grape seeds were obtained at local markets in Assiut, Egypt.

### 2.2. Preparation of Grape Seeds Powder

The grape seed samples were rinsed and left to dry after being cleaned with tap water. To make grape seeds flour, they were ground to a powder in a mortar and pestle and sieved with a mesh size of 0.50 mm. For further analysis, all dried powder samples were kept in clean brown bottles at room temperature.

### 2.3. Proximate Chemical Composition

Moisture, protein, fat, fiber, and ash contents of raw samples were measured using A.O.A.C [44] protocol. Total carbohydrates were measured using the protocol of Nwosu et al. [45] and using the following equation:

% carbohydrates = 100 − (% moisture + % protein + % ash + % fat + % fiber).

### 2.4. Determination of Total Phenols, Flavonoids, and Scavenging Activity

One gram of grape seed powder was mixed with 25 mL of methanol to prepare a methanolic extract. This mixture was left on the shaker for 24 h. Subsequently, it was centrifuged at 10,000 rpm for 15 min, and therefore, the supernatant was filtrated by Whatman No 41. The supernatant was diluted to 25 mL, then kept in the refrigerator (4 °C) and used for one week. Total phenol content was measured by using the Folin–Ciocalteu reagent according to the protocol of Wu et al. [46]. Flavonoid content was determined using Folin–Ciocalteu reagent and aluminum chloride according to Baba and Malik’s [47] method.

The DPPH assay was measured according to the protocol of Park et al. [48] and the absorbance was read at 515 nm using a spectrophotometer. The following formula was used to quantify scavenging activity:

$$\mathrm{DPPH}\;\mathrm{radical}\text{-}\mathrm{scavenging}\;\mathrm{activity}\;(\%)\;=\;\frac{\mathrm{Absorbance}\;\mathrm{of}\;\mathrm{control}-\mathrm{Absorbance}\;\mathrm{of}\;\mathrm{sample}}{\mathrm{Absorbance}\;\mathrm{of}\;\mathrm{control}}\;\times \;100$$

The ABTS assay was measured using Re et al.’s [49] protocol and the absorbance was read at 734 nm using a spectrophotometer. Scavenging activity was calculated using the following formula:

$$\mathrm{ABTS}\;\mathrm{radical}\text{-}\mathrm{scavenging}\;\mathrm{activity}\;(\%)\;=\;\frac{\mathrm{Absorbance}\;\mathrm{of}\;\mathrm{control}-\mathrm{Absorbance}\;\mathrm{of}\;\mathrm{sample}}{\mathrm{Absorbance}\;\mathrm{of}\;\mathrm{control}}\;\times \;100$$

### 2.5. Determination of Metal Elements

Minerals were estimated for samples of balady bread (Bb) control, grape seeds powder (GSP), and balady bread supplemented with different concentrations of GSP, using an atomic absorption device, according to the approved method [44].

### 2.6. Extraction of Polyphenolics of Grape Seeds

About 10 g of fresh ground grape seeds was extracted by soaking them in 50 mL of 99.9% Sigma HPLC grade methanol overnight at 25 °C in a dark bottle, then filtering the solvent (Whatman No.1) and re-soaking the solids in 50 mL of fresh methanol overnight and pooling the filtrate. The filtrate was concentrated under reduced pressure at 45 °C, and 2 mL of the extract was filtered through 0.22 µm PTFE and stored at −20 °C in a 1.5 mL Amber high-performance liquid chromatography (HPLC) vial until analysis.

### 2.7. HPLC Analysis of Phenolic Components

The phenolic compounds were separated using the method described by Yadav et al. [50]. Analyses were performed on a reverse-phase HPLC system (Dionex, Ultimate 3000, Sunnyvale, CA, USA) loaded with a diode array detector. The column was a reversed-phase column (Acclaim R-120, Dionex C-18, 5 lm, 120 A°, 4.6 9 250 mm, Sunnyvale, CA, USA). All phenolic compounds were identified at 277 nm. The solvent gradient was created by ranging the proportion of solvent A (1% acetic acid in water, *v*/*v*) to solvent B. (acetonitrile). The flow rate was set to 0.300 mL/min, and the column temperature to 30 °C. Grape seed powder was extracted using 10% (*v*/*v*) methanol in water, yielding a concentration of 1.0 mg/mL. All mixture was then filtered through nylon filters with pore sizes of 0.2 µm. The phenolic components in the sample extract were defined as compared to the retention time of the standards. 

### 2.8. Farinograph Properties

Farinograph properties of the flour and three levels (5, 10, and 15%, *w*/*w*) of grape seed powder supplemented flour samples were determined in line with an approved method [44] employing a farinograph equipped with a 300 g bowl.

### 2.9. Preparation of Grape Seeds Balady Bread

Balady bread (Bb) was prepared as described by Hegazy et al. [51] instructions. Balady bread was prepared by using grape seed flour in various doses (5%, 10%, and 15%) as a partial replacement for wheat flour. Table 1 shows the formulas for 85, 90, 95, and 100 g of wheat flour, 2 percent fresh yeast diluted in warm water (40 °C), 3.5 g of corn oil, 2 g salt, and 50–72 mL water.

The flour and other ingredients were combined, and the dough was allowed to ferment for 40 min at room temperature. The dough was turned into loaves and baked in an electric oven for 3 min at 250 °C. They were then cooled by air and sealed in polyethylene bags until they were used for analysis and measurements.

### 2.10. Sensory Evaluation Methods

Bread samples were sensorial evaluated after baking by 20 panelists who were staff and non-staff members of the Department of Home Economics, Faculty of Specific Education, Assiut University. All samples were provided in plates having white color at ambient temperature. The panelists were asked to evaluate each sample of the bread for taste, chewing ability, texture, aroma, color, roundness, crumb, appearance, and overall acceptability. The samples were rated on a 1–9-point hedonic scale (1 = dislike extremely, 2 = dislike very much, 3 = dislike moderately, 4 = dislike slightly, 5 = neither like nor dislike, 6 = like slightly, 7 = like moderately, 8 = like very much and 9 = like extremely) according to Stone and Sidel [52]. Scores were collated and analyzed statistically.

### 2.11. Statistical Analysis

The statistical analyses of the results were performed using the computerized SPSS program (Statistical Package for the Social Sciences). The effects of various treatments were analyzed using a one-way ANOVA (Analysis of variance) test with Duncan’s multiple range test [53].

## 3. Results and Discussions

### 3.1. Chemical Composition of Grape Seed Powder (GSP) and Balady Bread Supplemented with Different Ratios of GSP

The chemical analysis of grape seed powder (GSP), and balady bread (Bb) supplemented with different concentrations of GSP (5, 10, and 15%) was illustrated in Table 2. According to the findings, grape seed powder (GSP) has more ash 3.54%, fats 10.48%, protein 13.14%, carbohydrates 31.68%, and dietary fiber 34.0% than Bb control, which has less ash 1.9%, fats 3.55%, protein 12.05%, carbohydrates 29.83%, and dietary fiber 1.02%. These results are in agreement with those of Aghamirzaei et al. [28] who indicated that grape seeds contained 17.11% fat, 10.94% crude protein, 42.74% crude fiber, and 2.45% ash), but the content of moisture was about 7.48%. In addition, the results showed that the content of both iron (37.7 mg/kg), and zinc (9.27 mg/kg) in grape seed powder was greater than their content in the balady bread (Bb) control (8.3 and 6.52 mg/kg, respectively). The results obtained were less than those mentioned in the study of Mohamed et al. [54]. This difference may be due to the variety and the area of cultivation, as well as the part taken from the plant, whether it is the seed, the remaining after pressing, or the leaves. Finally, the composition of the soil and the type of fertilizer.

The results showed that increasing GSP levels led to an increase in the nutritional value of bread, including dietary fiber, fat, iron, and zinc. Mineral elements play an important role in the metabolism of the human body through the activation of many enzymatic systems of a physicochemical nature through pH control, electrical neutrality, and chemical potential gradients [55,56,57]. It was also noted from Table 2 that the content of both iron and zinc increased by increasing the percentage of replacing wheat flour with grape seed flour [54]. The chemical analysis showed that GSP is a completely functional food rich in basic nutrients.

The results showed that GPS contain high amounts of fat, protein, carbohydrate, and fibers (Table 2). Fiber-rich by-products can be commonly used in food products as low-cost, non-caloric bulking agents to substitute flour, fat, or sugar, as well as improving water and oil preservation to enhance emulsion or oxidative stability [58]. Besides their health benefits, dietary fibers have a variety of relevant characteristics such as water-holding capacity, swelling capacity, increasing viscosity, and gel formation, all of which are essential in the preparation of food products [59]. Severe protein insufficiency, as well as the rising costs of products of animal origin, has prompted research into developing new protein sources from underutilized wastes and by-products [60,61]. The use of grape leaves and seeds in the manufacturing of value-added protein supplements has the potential to reduce waste and thus environmental issues [8].

### 3.2. Phenolic Acids Identification and Antioxidants Capacity in a Grape Seed Powder

**Figure 1:**
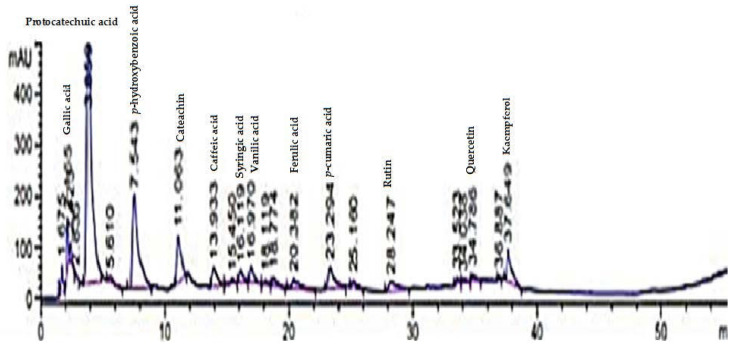
HPLC of different phenolic compounds in grape seed powder.

**Figure 2:**
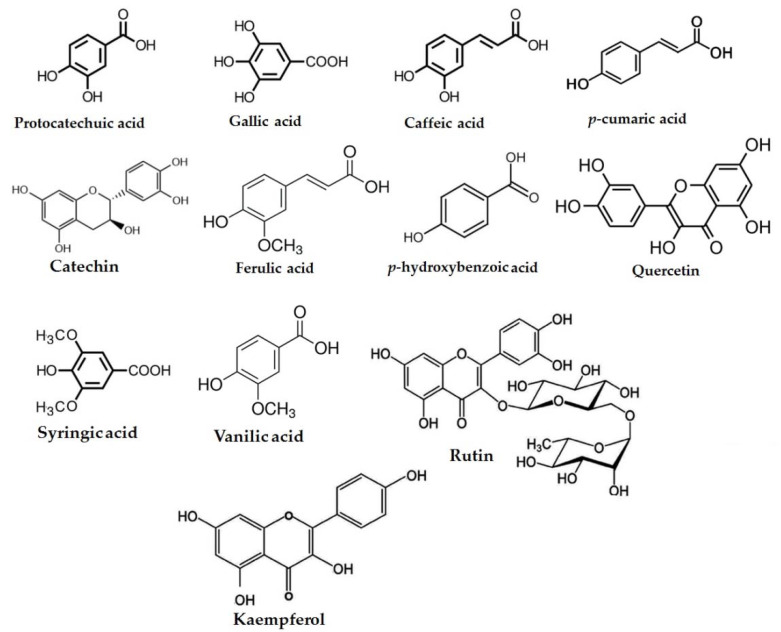
Chemical structure of phenolic compounds in grape seed powder extract.

The individual phenolic content determined by HPLC is presented in Table 3 and Figure 1 and Figure 2. The gallic, protocatechuic, and p-hydroxybenzoic acid contents were 23.87, 17.45, and 5.39 mg/g of grape seed powder, respectively. Among individual phenolic content, the amounts of gallic and protocatechuic were higher than other phenolic acids. These findings were consistent with those of Abdel-Khalek and Mattar [8] who discovered the presence of phenolic compounds in Egyptian grape leaves, grape seeds, and mulberry leaves. They discovered that phenolic compounds differed in the methanolic extracts of all samples. The major polyphenols in the three extracts were gallic acids, ferulic acids, catechins, chlorogenic acids, caffeic acids, and coumaric acids. The phenolic compounds in grapes have a broad range of biological activity as well as potential health benefits [9,62]. They have anti-inflammatory, anti-amyloidogenic, anti-cholinesterase, anti-amnesic, hypolipidemic, anti-aging, and immunomodulatory characteristics, and they are food products that can help slow the neurocognitive decline associated with aging and Alzheimer’s disease and promote human health [63]. Numerous clinical studies have emphasized the significance of phenolic acids in attempting to control a variety of human diseases, such as severe acute respiratory syndrome (SARS) and the Middle East respiratory syndrome (MERS), both of which are associated with COVID-19, by improving the human immune system to viral infections [64].

**Figure 3:**
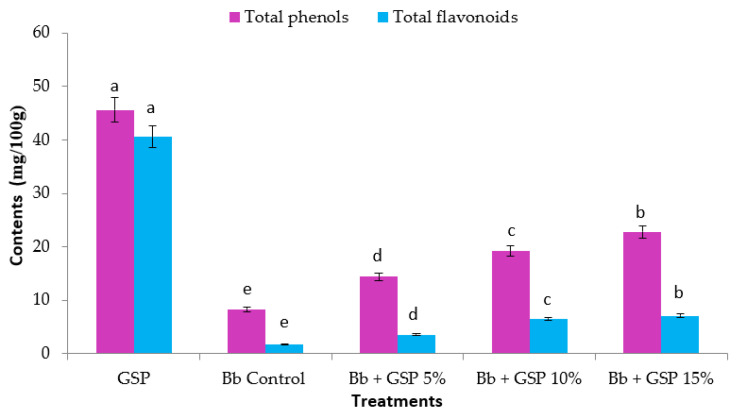
Total phenolic (mg GAE/100 g) and total flavonoids (mg QE/100 g) contents in balady bread (Bb) supplemented with different ratios of grape seed powder (GSP). Each value is a mean (±SD) of three replicates. The different letters on the same bar show a significant difference according to Duncan’s test at *p* ≤ 0.05. GAE: gallic acid; QE: quercetin.

Phenolic compounds are biologically active secondary metabolites observed in common edible plant foods [65,66]. The total phenolic content (TPC) and total flavonoid (TF) of GSP and balady bread (Bb) are given in Figure 3. GSP has a high concentration of TPC (approximately 45.6 mg GAE/100 g), whereas control bread has the lowest concentration of phenolics (approximately 8.3 mg GAE/100 g). The TPC value of bread ranged from 8.3 to 22.8 mg GAE/100 g. By increasing GSP concentrations, the total phenolic content of bread produced with GSP was substantially increased (Figure 3). TPC increased three-fold as GSP substitute increased from 0 to 15 g GSP/100 g flour. These results are in accordance with those described by Kupe et al. [67] who discovered that grape seeds contain higher phenolic content than peel and pulp, and that total phenolic content was about 245–207 mg GAE/100 g in nine cultivars of grape seeds. Many studies have shown that phenolic compounds have antioxidant capacity. TPC and antioxidant activity have positive correlations [68]. Phenolic compounds exhibit antioxidant activity by donating hydrogen, scavenging free radicals, acting as chelators, and quenching reactive oxygen species [68,69]. In the case of bread, phenolic compounds were discovered to have a protective effect against the formation of carcinogens during baking [70]. GSP contains high concentrations of TF with about 40.6 mg QE/100 g compared to Bb, which contains the lowest value of about 1.7 mg QE/100 g (Figure 3) The total flavonoid content of bread produced with the addition of different levels of GS ranged from 1.7 to 7.1 mg QE/100 g. Total flavonoids increased substantially as GS levels in bread increased. There are positive relations between total flavonoids and antioxidant capacity [68].

**Figure 4:**
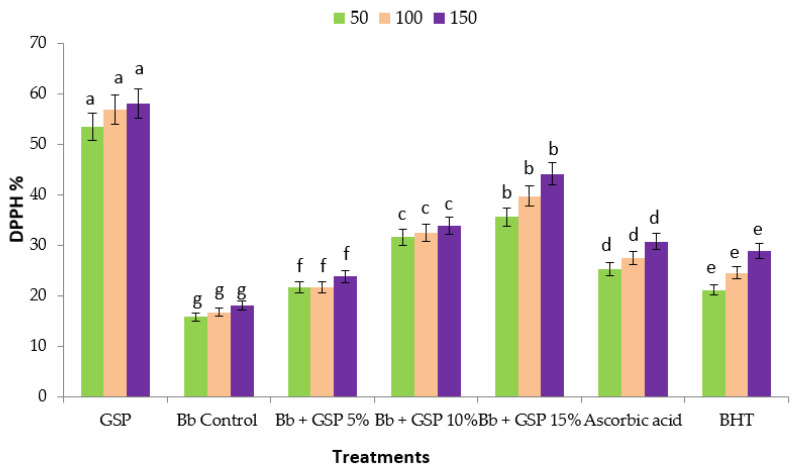
Antioxidants capacity % of balady bread (Bb) supplemented with different ratios of grape seed powder (GSP) extracts at different concentrations against DPPH. Each value is a mean (±SD) of three replicates. The different letters on the same bar show a significant difference according to Duncan’s test at *p* ≤ 0.05.

**Figure 5:**
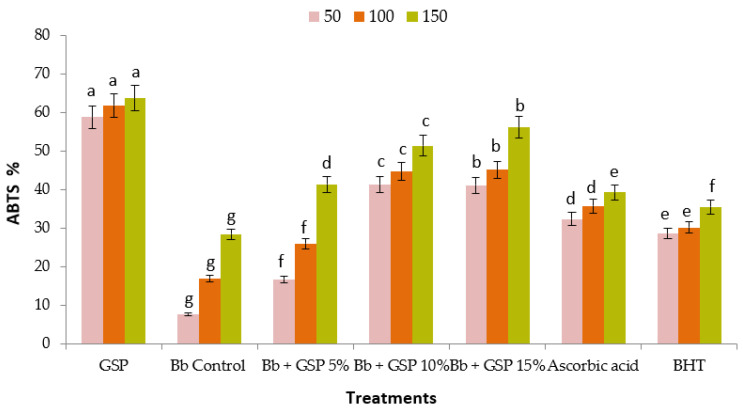
Antioxidants capacity % of balady bread (Bb) supplemented with different ratios of grape seed powder (GSP) extracts at different concentrations (µg/mL) against ABTS. Each value is a mean (±SD) of three replicates. The different letters on the same bar show a significant difference according to Duncan’s test at *p* ≤ 0.05.

The antioxidant activity of grape seed powder extracts and balady bread prepared with different levels of GSP was assessed using the DPPH free-radical scavenging activity, and the results are shown in Figure 4. The results demonstrated that GSP has a higher antioxidant capacity; the effect was related to the GSP levels. Grape seed extract had a higher antioxidant capacity than control bread (no additives). These findings agreed with those of Guaita and Bosso [71], who discovered that grape seeds contain a higher antioxidant capacity than grape peels, suggesting a relationship between tannin content and antioxidant capacity. The acetonic extract of grape seed had substantially higher total phenolic and flavonoid content, as well as the best DPPH scavenging capability of the extracts tested [72]. Moreover, the antioxidant activity in control bread was 29%. This activity was most likely caused by the Maillard reaction, as Maillard reaction products, particularly melanoidins, have antioxidant properties. The addition of 7.5% grape seed boosted the antioxidant capacity with radical inhibition reaching 69%. According to the findings, adding grape seed to bread boosted its antioxidant capacity. The incorporation of phenolic compounds may be responsible for the increased antioxidant activity of bread supplemented with grape seed [68]. The same trend was noticed with the ABTS test (Figure 5). These findings show that GSP fortified bread contains substantial quantities of phenolic compounds, which are nutritional effective. Phenolic antioxidants are a type of food-active component that can be used to add extra health advantages to a variety of value-added processed foods. At 2.5, 5, and 7.5 g GSF/100 g WF, the bread antioxidant activity increased significantly [37].

### 3.3. Rheological Properties of Balady Bread Supplemented with Different Ratios of GSP

**Figure 6:**
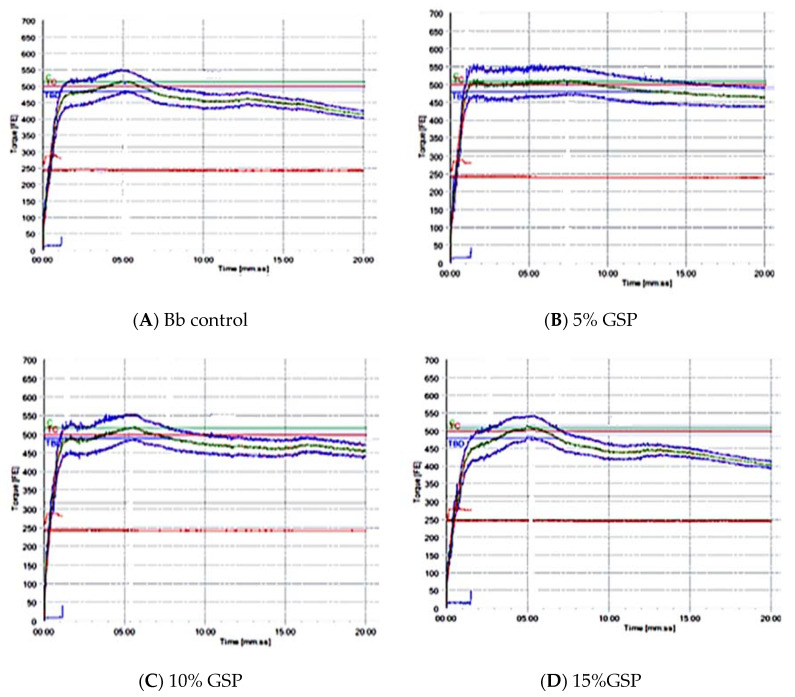
Farinogram for wheat flour and replacement ratios for grape seed powder.

The rheological properties of the doughs of wheat flour substituted with 5%, 10%, and 15% of grape seed powder and their effects on most of the measurements were studied for the farinograph test. The rheological properties of the dough are important because they affect both the mechanical ability of the dough and the quality of the final product, as shown in Table 4 and Figure 6. 

It is noted from Table 4 and Figure 6 that there are significant differences (*p* ≤ 0.05) in the absorption ratio of samples of flour to which grape seeds have been added. When replacing wheat flour with grape seed powder, the water absorption rate increased with increasing addition level of GSP, and this is in agreement with what was mentioned by Tolve et al. [73] who studied the effect of adding grape seed flour on the rheological characteristics of the dough, and they found that the amount of water absorbed by the dough increased with the increase in the replacement ratios of 0, 5, and 10% of grape seeds, which amounted to 55.50%, 56.80%, and 60.03%, respectively. This increase is related to a higher content of dietary fibers in the dough after adding grape seed flour, as noted by Mironeasa et al. [74]. The dietary fiber has a high number of hydroxyl groups, which allows for more interactions with water molecules via hydrogen bonds.

The results of Table 4 and Figure 6 show that there are significant differences (*p* ≤ 0.05) between the dough stability time for wheat flour and samples of wheat flour substituted with different percentages of grape seed powder, as well as significant differences (*p* ≤ 0.05) between the replacement percentages of grape seed powder. As the dough stability period for wheat flour reached 4.99 min, the dough stability period increased significantly for grape seed powder replacement rates of 5% and 10%, reaching 12.72 and 7.18 min, respectively. These results are in accordance with those of Aghamirzaei et al. [28], who studied the effect of adding grape seed flour on the properties of wheat flour with the replacement ratios of 5%, 10%, 15%, 20%, and 25%. They found that the highest stability was at a replacement level of 10%, and the higher the replacement rate, the less stable the dough. It is due to the presence of fatty compounds in grape seed powder, which interfere with the polymeric part of gluten, leading to the softening of the dough and improving the rheological properties of the dough. This could be due to the formation of the gluten network and dough stabilization in the rheological properties of the dough. Other research has found that replacing WF with GSP at levels of 3, 5, and 7 g GSP/100 g. WF reduces the falling number (FN) index, increases alpha-amylase activity in wheat-grape seed composite flour, and improves dough rheological properties all at the same time [74]. GSP addition helped to improve crop value and the nutritional value of bakery products [75], resulting in better harmony of antioxidant properties, sensory quality, and consumer acceptability [76].

Table 4 data also revealed that dough development time, which is the time required to reach maximum peak viscosity, is an indicator of flour quality. The time required for dough development ranged from 4.95 to 8.71 min. GSP at levels of 15% had no effect on dough development time, but GSP at levels of 5.0 and 10% improved dough development time. The increment in dough development time indicates that the flour is stronger. Flour strength is indicated by the stability value and the mixing tolerance index, with higher stability and lower mixing tolerance index values indicating stronger doughs. The dough stability value increased as the level of GSP increased from 0% to 10%. The highest dough stability value was detected at 5% GSP by approximately 12.72 min, and the mixing tolerance index was reduced by approximately 33.45 BUE. Dough strength and weakness are critical aspects of baking. Strong dough is commonly preferred in most baking applications due to its superior rheological and handling characteristics, as well as its superior form and texture characteristics. Bakers have used oxidants to strengthen dough for centuries. These oxidants help form disulfide bonds, which strengthen the dough [68]. The results of this study’s rheological tests revealed that GSP had a significant strengthening effect on dough. The stability values of GSP-containing dough elevated, while the MTI value reduced. The hydroxyl groups in phenolic compounds may be responsible for this strengthening effect. GSP contains a high concentration of phenolic compounds [68]. Phenolic compounds not only influence the oxidative reactions of food proteins but can also interact with proteins directly, which tends to result in noncovalent or covalent bonding. Protein–polyphenol interactions alter proteins, influencing the quality and functional properties of food [77]. Furthermore, higher protein levels in wheat flours replaced with 5% GSP may contribute to improved dough stability. In terms of GSP proteins, increased dough stability in samples with 5% substitution GSP could be attributed to protein aggregation [78].

### 3.4. Sensory Properties of Balady Bread Supplemented with Different Ratios of GSP

**Figure 7:**
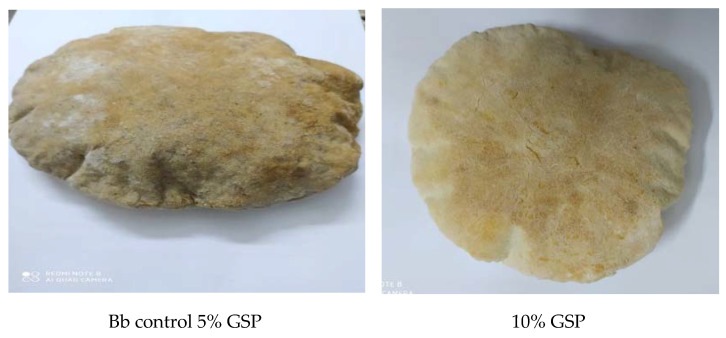

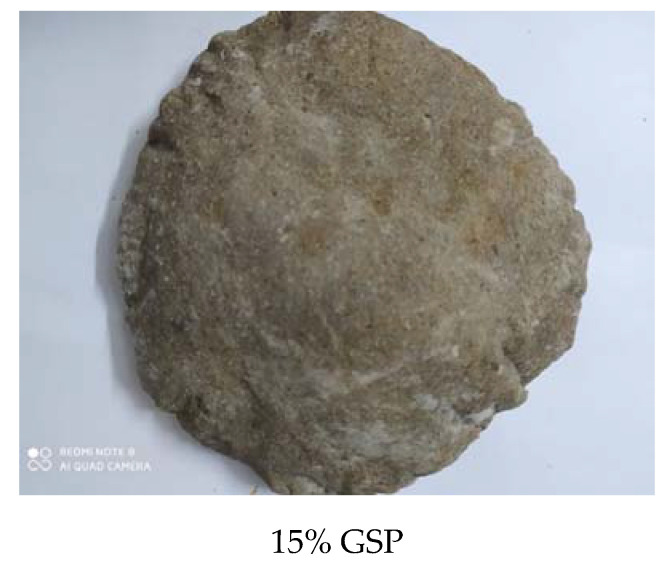
Balady bread supplemented with different ratios of GSP.

The data in Table 5 and Figure 7 showed the sensory characteristics of balady bread such as the organoleptic characteristics (taste, chewing ability, texture, aroma, color, crumbs, appearance, and overall acceptability) of control and grape seed powder-enriched bread samples tested on 20 panelists. The panelists gave the control sample (bread made from wheat flour with 72% extraction) the highest scores for all sensory characteristics. On the contrary, balady bread samples incorporated with grape seed powder at 15% were scored below the acceptability range. For all measured characteristics, there were no significant differences between the bread samples made from wheat flour incorporated with GSP 5% and 10%. Balady bread samples incorporated with grape seed powder at 10% scored less than the control but were still acceptable. The bread crumbs supplemented with GSP were all darker than the control, and the crumb value was reduced by increasing the addition of GSP. These results are in accordance with Meral and Doğan [68]. Catechin and epicatechin are phenolic compounds found in GSP [8]. These compounds have an impact on the color of products.

Panelist scores for general appearance decreased significantly as GSP supplementation levels increased by more than 10%, as did scores for chewing ability, roundness, and crumb. An increase in the fiber content in bread leads to a weakening of the gluten network, and therefore its ability to retain the gases that formed and expanded during oven spring decreases [79]. These results are in accordance with those obtained by previous studies [80,81,82]. 

Several studies have been conducted to examine the effects of various levels of grape seed flour (GSP) supplementation on dough rheological behavior and bread quality. For example, replacing WF with up to 10% GSF increased the nutritional value of bakery products without affecting dough rheological or sensorial bread properties [75].

## 4. Conclusions

Bread is one of the most popular foods in the world. There is an increasing demand for new healthier bread products that are also high in quality. Grape seeds are a great source of fiber, iron, and zinc. The effect of integrating GSP on flour quality attributes and rheological characteristics was investigated in this study. GSP addition in wheat flour reduced total carbohydrates while increasing total dietary fiber, fat, and total phenol. The addition of GSP to wheat flour reduced development time, farinograph quality number, and dough stability in farinograph above 10%, while increasing water absorption and mixing tolerance. The GSP-blended dough’s physicochemical properties and farinograph results revealed that a maximum of 10% GSP could be integrated into the flour to obtain satisfactory physical properties. For all measured sensory characteristics, there were significant differences between the bread samples made from wheat flour incorporated with GSP 5% and the control sample. Balady bread samples incorporated with grape seed powder at 10% scored less than the control but were still acceptable. The replacement of WF with up to 10% GSF adds value and increases the nutritional value of bakery products. This will lead to a good balance of high antioxidant activity, dietary fiber, iron, and zinc content with consumer acceptability.

## Figures and Tables

**Table 1:** Balady bread (Bb) with different concentrations of grape seeds powder (g/100 g).

Ingredients	Bb Control	GSP 5%	GSP 10%	GSP 15%
wheat flour 72% (g)	100	95	90	85
Grape seeds flour (g)	0	5	10	15
Corn oil	3.5	3.5	3.5	3.5
Sugar	6	6	6	6
Salt (g)	2	2	2	2
Water (mL)	60	66	72	79
Yeast	3	3	3	3

GSP: grape seeds powder.

**Table 2:** Chemical composition of grape seed powder (GSP), balady bread (Bb) control, and balady bread supplemented with different concentrations of GSP.

Samples	Moisture (%)	Ash (%)	Fat (%)	Protein (%)	Total Carbohydrate (%)	Fiber (%)	Fe (mg/kg)	Zn (mg/kg)
GSP	7.16 ± 0.85 ^d^	3.54 ± 0.17 ^a^	10.48 ± 0.58 ^a^	13.14 ± 0.31 ^b^	31.68 ± 0.53 ^a^	34.0 ± 0.83 ^a^	37.7 ± 0.51 ^a^	9.27 ± 0.32 ^a^
Bb control	51.65 ± 0.71 ^c^	1.9 ± 0.21 ^c^	3.55± 0.26 ^d^	12.05 ± 0.40 ^c^	29.83 ± 0.33 ^b^	1.02 ± 0.16 ^e^	8.3 ± 0.27 ^e^	6.52 ± 0.34 ^d^
GSP 5%	53.72 ± 0.46 ^b^	3.08 ± 0.31 ^b^	4.83 ± 0.35 ^c^	13.82 ± 0.41 ^a^	21.20± 0.57 ^c^	3.35 ± 0.3 ^d^	13.15 ± 0.37 ^d^	7.16 ± 0.16 ^c^
GSP 10%	54.8 ± 0.56 ^a^	3.41 ± 0.25 ^a^	6.75 ± 0.55 ^b^	13.85 ± 0.38 ^a^	16.24 ± 0.61 ^d^	4.95 ± 0.48 ^c^	14.96± 0.44 ^c^	7.79 ± 0.25 ^b^
GSP 15%	55.19 ± 0.26 ^a^	3.48 ± 0.27 ^a^	7.55 ± 0.62 ^b^	13.63 ± 0.26 ^a^	13.25. ± 0.72 ^e^	6.9 ± 0.50 ^b^	21.51 ± 0.5 ^b^	7.61 ± 0.19 ^b^

Each value is a mean (±SD) of three replicates. The different letters on the same bar show a significant difference according to Duncan’s test at *p* ≤ 0.05.

**Table 3:** Phenolic content in grape seed powder extract.

Compound	Concentration (mg/g)
Gallic acid	23.87
Protocatechuic acid	17.45
*p*-hydroxybenzoic acid	5.39
Cateachin	1.39
Caffeic acid	1.49
Syringic acid	0.61
Vanilic acid	0.82
Ferulic acid	1.06
*p*-cumaric acid	0.11
Rutin	3.58
Quercetin	0.18
Kaempferol	0.13

**Table 4:** Farinograph parameters of balady bread dough supplemented with different ratios of GSP.

Samples	Water Absorption (WA) %	Dough Development Time (DTT) Min	Dough Stability Min	Mixing Tolerance Index (MTI) BUE *	Farinograph Quality Number	Time to Breakdown
Bb control	63.66 ± 0.53 ^c^	4.59 ± 0.52 ^c^	4.99 ± 0.72 ^c^	69.46 ± 1.59 ^b^	68.46 ± 1.41 ^c^	6.03 ± 1.52 ^b^
GSP 5%	65.85 ± 0.48 ^b^	8.71 ± 0.48 ^a^	12.72 ± 0.79 ^a^	33.45 ± 1.48 ^d^	129.45 ± 1.48 ^a^	13.95 ± 1.45 ^a^
GSP 10%	66.89 ± 0.38 ^a^	6.14 ± 0.37 ^b^	7.18 ± 0.51 ^b^	56.89 ± 0.78 ^c^	79.89 ± 0.78 ^b^	8.57 ± 0.70 ^b^
GSP 15%	67.26± 0.41 ^a^	5.12 ± 0.62 ^c^	4.09 ± 0.81 ^c^	80.06 ± 1.91 ^a^	68.06± 1.91 ^c^	6.53 ± 1.91 ^b^

Each value is a mean (±SD) of three replicates. The different letters on the same column show a significant difference according to Duncan’s test at *p* ≤ 0.05. * Brabender units equivalent.

**Table 5:** Sensory evaluation of balady bread dough supplemented with different ratios of GSP.

Samples	Taste	Chewing Ability	Texture	Aroma	Color	Roundness	Crumb	Appearance	Overall Acceptability
Bb control	9.4 ± 0.70 ^a^	9.0 ± 1.05 ^a^	9.1 ± 0.54 ^a^	9.2 ± 0.59 ^a^	9.2 ± 0.69 ^a^	9.2 ± 0.55 ^a^	9.3 ± 0.67 ^a^	9.0 ± 0.72 ^a^	9.3 ± 0.67 ^a^
GSP 5%	7.6 ± 0.51 ^b^	8.1 ± 1.20 ^ab^	7.7 ± 1.16 ^b^	7.8 ± 1.03 ^b^	7.8 ± 0.73 ^b^	7.1 ± 1.16 ^b^	7.9 ± 0.59 ^b^	7.8 ± 1.05 ^b^	8.5 ± 0.75 ^ab^
GSP 10%	7.2 ± 0.32 ^b^	7.2 ± 1.14 ^b^	6.9 ± 1.20 ^b^	7.8 ± 1.32 ^b^	6.6 ± 0.85 ^c^	6.2 ± 1.03 ^bc^	6.4 ± 0.46 ^c^	6.9 ± 1.12 ^b^	7.6 ± 0.57 ^b^
GSP 15%	5.2 ± 0.83 ^c^	5.2 ± 0.72 ^c^	5.3 ± 0.64 ^c^	5.8 ± 0.42 ^c^	5.8 ± 0.43 ^c^	5.2 ± 0.32 ^c^	5.6 ± 0.35 ^d^	5.1 ± 0.40 ^c^	5.9 ± 0.52 ^c^

Each value is a mean (±SD) of three replicates. The different letters on the same column show a significant difference according to Duncan’s test at *p* ≤ 0.05.

## Data Availability

Data is contained within the article.

## References

[1] Bruno J.A., Konas D.W., Matthews E.L., Feldman C.H., Pinsley K.M., Kerrihard A.L. (2019). Sprouted and non-sprouted chickpea flours: Effects on sensory traits in pasta and antioxidant capacity. Pol. J. Food Nutr. Sci..

[2] Cappa C., Alamprese C. (2017). Brewer’s spent grain valorization in fiber-enriched fresh egg pasta production: Modelling and optimization study. LWT-Food Sci. Technol..

[3] Drabińska N., Ciska E., Szmatowicz B., Krupa-Kozak U. (2018). Broccoli by-products improve the nutraceutical potential of gluten-free mini sponge cakes. Food Chem..

[4] El-Beltagi H.S., El-Mogy M.M., Parmar A., Mansour A.T., Shalaby T.A., Ali M.R. (2022). Phytochemical characterization and utilization of dried red beetroot (*Beta vulgaris*) peel extract in maintaining the quality of *Nile Tilapia* Fish Fillet. Antioxidants.

[5] Majzoobi M., Poor Z.V., Jamalian J., Farahnaky A. (2016). Improvement of the quality of gluten-free sponge cake using different levels and particle sizes of carrot pomace powder. Int. J. Food Sci. Technol..

[6] Abu-Shahba M.S., Mansour M.M., Mohamed H.I., Sofy M.R. (2022). Biosorptive Removal of Cadmium Ions from hydroponic Solution with indigenous garlic peel and mercerized garlic peel on lettuce productivity. Sci. Hortic..

[7] Ramón-Gonçalves M., Gómez-Mejía E., Rosales-Conrado N., León-González M.E., Madrid Y. (2019). Extraction, identification and quantification of polyphenols from spent coffee grounds by chromatographic methods and chemometric analyses. J. Waste Manag..

[8] Abdel-Khalek H.H., Mattar Z.A. (2022). Biological activities of Egyptian grape and mulberry by-products and their potential use as natural sources of food additives and nutraceuticals foods. J. Food Meas. Charact..

[9] Faustino M., Veiga M., Sousa P., Costa E.M., Silva S., Pintado M. (2019). Agro-food byproducts as a new source of natural food additives. Molecules.

[10] Ferreira-Santos P., Zanuso E., Genisheva Z., Rocha C.M.R., Teixeira J.A. (2020). Green and sustainable valorization of bioactive phenolic compounds from pinus by-products. Molecules.

[11] Carocho M., Barreiro M.F., Morales P., Ferreira I.C., Gomez P.M. (2014). Adding molecules to food, pros and cons: A review on synthetic and natural food additives. Compr. Rev. Food Sci. Food Saf..

[12] Krupa-Kozak U., Drabi´nska N., Baczek N., Šimková K., Starowicz M., Jeli´nski T. (2021). Application of Broccoli Leaf Powder in Gluten-Free Bread: An Innovative Approach to Improve Its Bioactive Potential and Technological Quality. Foods.

[13] Pfukwa T.M., Fawole O.A., Manley M., Pieter A., Gouws P.A., Opara U.L., Mapiye C. (2019). Food preservative capabilities of grape (*Vitis vinifera*) and clementine mandarin (*Citrus reticulata*) byproducts extracts in South Africa. Sustainability.

[14] Du C., Abdullah J.J., Greetham D., Fu D., Yu M., Ren L., Li S., Lu D. (2018). Valorization of food waste into biofertiliser and its field application. J. Clean. Prod..

[15] Shirahigue L.D., Regina S., Antonini C. (2020). Agro-industrial wastes as sources of bioactive compounds for food and fermentation industries. Food Technol..

[16] Jaisanthi J., Thahira A. (2014). Phytonutrient composition, antioxidant activity and acceptability of baked product incorporated with grape seed extract. J. Hum. Nutr. Food Sci..

[17] Jordão A.M., Cosme F. (2018). Grapes and Wines—Advances in Production, Processing, Analysis and Valorization.

[18] Peixoto C.M., Inês M., Alves M.J., Calhelha R.C. (2018). Grape pomace as a source of phenolic compounds and diverse bioactive properties. Food Chem..

[19] Poveda J.M., Loarce L., Alarcón M.A. (2018). Revalorization of winery by-products as source of natural preservatives obtained by means of green extraction techniques. Ind. Crops Prod..

[20] Hoye C., Ross C. (2011). Total phenolic content, consumer acceptance, and Instrumental analysis of bread made with grape seed flour. J. Food Sci..

[21] Peighambardoust S., Aghamirzaei M. (2014). Physicochemical, nutritional, shelf life and sensory properties of iranian sangak bread fortified with grape seed powder. J. Food Process Technol..

[22] Burcinl E., Vural H. (2011). Grape seed four is a viable ingredient to improve the nutritional profile and reduce lipid oxidation of frankfurters. Meat Sci..

[23] Rubilar J., Cruz R., Khmelinskii I., Vieira M. (2013). Effect of antioxidant and optimal antimicrobial mixtures of carvacrol, grape seed extract and chitosan on different spoilage microorganisms and their application as coatings on different food matrices. Int. J. Food Stud..

[24] Garcia M.M., Rivas-Gonzalo J.C., Ibanez E., Garcia M.C. (2006). Recovery of catechins and proanthocyanidins from winery by-products using subcritical water extraction. Anal. Chim. Acta..

[25] Choi C., Chung H., Choi M., Kang M. (2010). Effects of grape pomace on the antioxidant defense system in diet-induced hypercholesterolemic rabbits. Nutr. Res. Pract..

[26] Galanakis C.M., Aldawoud T.M.S., Rizou M., Rowan N.J., Ibrahim S.A. (2020). Food ingredients and active compounds against the Coronavirus disease (COVID-19) pandemic: A comprehensive review. Foods.

[27] Aksoylu Z., Cagindi O., Kose E. (2015). Effects of blueberry, grape seed powder and poppy seed incorporation on physicochemical and sensory properties of biscuit. J. Food Qual..

[28] Aghamirzaei M., Peighambardoust S.H., Azadmard-Damirchi S., Majzoob M. (2015). Effects of grape seed powder as a functional ingredient on flour physicochemical characteristics and dough rheological properties. J. Agric. Sci. Technol..

[29] Özcan M.M. (2010). Mineral contents of several grape seeds. Asian. J. Chem..

[30] Walker R., Tseng A., Cavender G., Ross A., Zhao Y. (2014). Physicochemical, nutritional, and sensory qualities of wine grape pomace fortified baked goods. J. Food Sci..

[31] Fernández-Fernández A.M., Dellacassa E., Nardin T., Larcher R., Ibañez C., Terán D., Gámbaro A., Medrano-Fernandez A., del Castillo M.D. (2022). Tannat Grape skin: A Feasible ingredient for the formulation of snacks with potential for reducing the risk of diabetes. Nutrients.

[32] Maner S., Sharma A.K., Banerjee K. (2017). Wheat flour replacement by wine grape pomace powder positively affects physical, functional and sensory properties of cookies. Proc. Natl. Acad. Sci. USA.

[33] Guiné R.P., Florença S.G., Barroca M.J., Anjos O. (2020). The link between the consumer and the innovations in food product development. Foods.

[34] Granato D., Barba F.J., Bursać Kovačević D., Lorenzo J.M., Cruz A.G., Putnik P. (2020). Functional foods: Product development, technological trends, efficacy testing, and safety. Annu. Rev. Food Sci. Technol..

[35] Abdel-Rahim E.A., El-Beltagi H.S. (2010). Constituents of apple, parsley and lentil edible plants and their therapy treatments for blood picture as well as liver and kidney functions against lipidemic disease. Elec. J. Environ. Agric. Food Chem..

[36] Afify A.E.-M.M.R., El-Beltagi H.S., Aly A.A., El-Ansary A.E. (2012). Antioxidant enzyme activities and lipid peroxidation as biomarker for potato tuber stored by two essential oils from Caraway and Clove and its main component carvone and eugenol. Asian Pac. J. Trop. Biomed..

[37] Acun S., Gul H. (2014). Effects of grape pomace and grape seed flours on cookie quality. Qual. Assur. Saf. Crop..

[38] El-Beltagi H.S., El-Desouky W., Yousef R.S. (2016). Synergistic antioxidant scavenging activities of grape seed and green tea extracts against oxidative stress. Not. Bot. Horti Agrobot. Cluj-Napoca.

[39] Helou C., Gadonna-Widehem P., Robert N., Branlard G., Thebault J., Librere S., Jacquot S., Mardon J., Piquet-Pissaloux A., Chapron S. (2016). The impact of raw materials and baking conditions on Maillard reaction products, thiamine, folate, phytic acid and minerals in white bread. Food Funct..

[40] Bredariol P., Vanin F.M. (2021). Bread Baking Review: Insight into Technological Aspects in Order to Preserve Nutrition. Food Rev. Int..

[41] Soliman A.S., Abbas M.S., Abol-Ella M.F., Eassawy M.M., Mohamed R.H. (2019). Towards bridging wheat gap in Egypt by using cassava, quinoa and guar as supplements for the production of balady bread. J. Food Meas. Charact..

[42] Yaseen A.A., Shouk A.E.H.A., Selim M.M. (2007). Egyptian balady bread and biscuit quality of wheat and triticale flour blends. Pol. J. Food Nutr. Sci..

[43] Ghonaim M.M., Mohamed H.I., Omran A.A.A. (2021). Evaluation of wheat salt stress tolerance using physiological parameters and retrotransposon-based markers. Genet. Resour Crop Evol..

[44] AOAC (2000). American of Cereal Association Chemists Approved Method of the AOAC.

[45] Nwosu J.N., Owuamanam C.I., Omeire G.C., Eke C.C. (2014). Quality parameters of bread produced from substitution of wheat flour with cassava flour using soybean as an improver. Am. J. Res. Comm..

[46] Wu C.-H., Murthy H.N., Hahn E.-J., Paek K.-Y. (2007). Improved production of caffeic acid derivatives in suspension cultures of Echinacea purpurea by medium replenishment strategy. Arch. Pharmacal Res..

[47] Baba S.A., Malik S.A. (2015). Determination of total phenolic and flavonoid content, antimicrobialand antioxidant activity of a root extract of *Arisaema jacquemontii* Blume. J. Taibah Univ. Sci..

[48] Park J.H., Lee Y.J., Kim Y.H., Yoon K.S. (2017). Antioxidant and antimicrobial activities of Quinoa (*Chenopodium quinoa* Willd.) seeds cultivated in Korea. Prev. Nutr Food Sci..

[49] Re R., Pellegrini N., Proteggente A., Pannala A., Yang M., Rice-Evans C. (1999). Antioxidant activity applying an improved ABTS radical cation decolourisation assay. Free Rad. Biol. Med..

[50] Yadav K., Bajaj R.K., Mandal S., Saha P., Mann B. (2018). Evaluation of total phenol content and antioxidant properties of encapsulated grape seed extract in yoghurt. Int. J. Dairy Technol..

[51] Hegazy A.L., Ammer M.S., Ibrahium M.I. (2009). Production of Egyptian gluten-free bread. World J. Dairy Food Sci..

[52] Stone H., Sidel J.L. (1993). The role of sensory evaluation in the food industry. Food Qual. Prefer..

[53] Bradley E.L., Blackwood L.G. (1989). Comparing paired data: A simultaneous test for means and variances. Am. Stat..

[54] Mohamed A.I.A., Özcan M.M., Al Juhaimi F., Babiker E.F.E., Ghafoor K., Banjanin T., Osman M.A., Gassem M.A., Alqah H.A. (2020). Chemical composition, bioactive compounds, mineral contents, and fatty acid composition of pomace powder of different grape varieties. J. Food Process. Preserv..

[55] Lachman J., Hejtmánková A., Hejtmánková K., Horníčková Š., Pivec V., Skala O., Dědina M., Přibyl J. (2013). Towards complex utilisation of winemaking residues: Characterisation of grape seeds by total phenols, tocols and essential elements content as a by-product of winemaking. Ind. Crops Prod..

[56] Shallan M.A., El-Beltagi H.S., Mona A.M., Amera T.M. (2010). Chemical evaluation of pre-germinated brown rice and whole grain rice bread. Elec. J. Environ. Agricult. Food Chem..

[57] El-Beltagi H.S., El-Senousi N.A., Ali Z.A., Omran A.A. (2017). The impact of using chickpea flour and dried carp fish powder on pizza quality. PLoS ONE.

[58] Elleuch M., Bedigian D., Roiseux O., Besbes S.C., Christophe B., Attia H. (2011). Dietary fiber and fiber-rich by-products of food processing: Characterization, technological functionality and commercial applications: A review. Food Chem..

[59] Ajila C.M., Aalami M., Leelavathi K., Prasada Rao U.J.S. (2010). Mango peel powder: A potential source of antioxidant and dietary fiber in macaroni preparations. Innov. Food Sci. Emerg. Technol..

[60] Lonnie M., Hooker E., Brunstrom J.M. (2018). Protein for life: Review of optimal protein intake, sustainable dietary sources and the effect on appetite in ageing adults. Nutrients.

[61] Abdel-Rahim E.A., El-Beltagi H.S., Romela R.M. (2013). White Bean seeds and Pomegranate peel and fruit seeds as hypercholesterolemic and hypolipidemic agents in albino rats. Grasas Y Aceites.

[62] Chen S.X., Ni Z.J., Thakur K., Wang S., Zhang J.G., Shang Y.F., Wei Z.J. (2021). Effect of grape seed power on the structural and physico chemical properties of wheat gluten in noodle preparation system. Food Chem..

[63] Fouad G.I., Rizk M.Z. (2019). Possible neuromodulating role of different grape (*Vitis vinifera* L.) derived polyphenols against Alzheimer’s dementia: Treatment and mechanisms. Bull. Natl. Res. Cent..

[64] Khalil A., Tazeddinova D. (2020). The upshot of polyphenolic compounds on immunity amid COVID-19 pandemic and other emerging communicable diseases: An appraisal. Nat. Prod. Bioprospecting..

[65] El-Beltagi H.S., Mohamed H.I., Abdelazeem A.S., Youssef R., Safwat G. (2019). GC-MS analysis, antioxidant, antimicrobial and anticancer activities of extracts from *Ficus sycomorus* fruits and leaves. Not. Bot. Horti Agrobot. Cluj-Napoca.

[66] Afify A.E.M.M.R., Shalaby E.A., El-Beltagi H.S. (2011). Antioxidant activity of aqueous extracts of different caffeine products. Not. Bot. Horti Agrobot. Cluj-Napoca.

[67] Kupe M., Karatas N., Unal M.S., Ercisli S., Baron M., Sochor J. (2021). Phenolic composition and antioxidant activity of peel, pulp and seed extracts of different clones of the turkish grape cultivar. Karaerik Plants.

[68] Meral R., Doğan İ.S. (2013). Grape seed as a functional food ingredient in bread-making. Int. J. Food Sci. Nutr..

[69] Abdel-Rahim E., El-Beltagi H.S., Ali R.F.M., Amer A.A., Mousa S.M. (2019). The effects of using synthetic and natural color foods on lipid profile and liver function in rats. Not. Bot. Horti Agrobot. Cluj-Napoca.

[70] Xu J., Wang W., Li Y. (2019). Dough properties, bread quality, and associated interactions with added phenolic compounds: A review. J. Funct. Foods.

[71] Guaita M., Bosso A. (2019). Polyphenolic characterization of grape skins and seeds of four Italian red cultivars at harvest and after fermentative maceration. Foods.

[72] Matloub A.A. (2018). Optimization of polyphenol extraction from *Vitis vinifera* L. leaves, antioxidant activity and its correlation with amelioration effect on AlCl_3_-induced Alzheimer’s disease. Arch. Pharm. Sci. Ain Shams Univ..

[73] Tolve R., Simonato B., Rainero G., Bianchi F., Rizzi C., Cervini M., Giuberti G. (2021). Wheat bread fortification by grape pomace powder: Nutritional, technological, antioxidant, and sensory properties. Foods.

[74] Mironeasa S., Codina G., Mironeasa C. (2012). The effect of wheat flour substitution with grape seed flour on the rheological parameters of the dough assessed by Mixolab. J. Texture Stud..

[75] Munteanu M.F., Gligor R., Alexa E., Poiana A.M., Onet M. (2013). Determination of the nutritional properties from grape seed flour. Curr. Opin. Biotechnol..

[76] Rosales Soto M.U., Brown K., Ross C.F. (2012). Antioxidant activity and consumer acceptance of grape seed flour-containing food products. Int. J. Food Sci. Technol..

[77] Baxter N.J., Lilley T.H., Haslam E., Williamson M.P. (1997). Multiple interactions between polyphenols and a salivary proline-rich protein repeat result in complexation and precipitation. Biochem..

[78] Valkova V., Duranova H., Stefanikova J., Miskeje M., Tokar M., Gabriny L., Kowalczewski P.Ł., Kacániova M. (2020). Wheat bread with grape seeds micropowder: Impact on dough rheology and bread properties. Appl. Rheol..

[79] Schleibinger M., Meyer A.L., Afsar N., Gyorgy N.A., Dicker V., Schmitt J.J. (2013). Impact of dietary fibers on moisture and crumb firmness of brown bread. Advnced J. Food Sci. Technol..

[80] Seleem H.A., Omran A.A. (2014). Evaluation quality of one layer flat bread supplemented with beans and sorghum baked on hot metal surface. Food Nutr. Sci.

[81] Eshak N.S. (2016). Sensory evaluation and nutritional value of balady flat bread supplemented with banana peels as a natural source of dietary fiber. Ann. Agric. Sci..

[82] Amer A.A., El-Beltagi H.S., Ali R.F.M., Mousa S.M., Abdel-Rahim E. (2019). The effects of wheat flour and barley flour on the quality and properties of biscuits colored with synthetic and natural colorants. Not. Bot. Horti Agrobot. Cluj-Napoca.

